# Supplementary material for: Neuropathologist-level integrated classification of adult-type diffuse gliomas using deep learning from whole-slide pathological images
Source: Nat Commun. 2023 Oct 11;14:6359. doi: 10.1038/s41467-023-41195-9 (PMC10567721; doi:10.1038/s41467-023-41195-9)
Supplement: Supplementary file 1 — Supplementary Information [file 41467_2023_41195_MOESM1_ESM.pdf]

Supplementary Information for

Neuropathologist-level integrated classification of adult-type diffuse gliomas using deep learning from whole-slide pathological images

Contents

Supplementary Methods

- A1: Patient recruitment and dataset description
- A2: Detection of IDH mutations and TERT promoter mutations by Sanger sequencing
- A3: Detection of Chromosome 1p/19q, CDKN2A, EGFR, and chromosome 7/10 status by Fluorescence in Situ Hybridization (FISH)
- A4: 2021 WHO Classification of adult-type diffuse gliomas in our datasets
- A5: The patch clustering algorithm
- A6: Deep learning network architecture and training scheme

Supplementary Figure 1-15

Supplementary Table 1-3

Supplementary Methods

A1: Patient recruitment

In total 2624 patients were included in this study and can be divided into three datasets. Dataset 1 included 1991 patients for developing and internally validating the deep learning classification model. Dataset 2 included 305 patients and Dataset 3 included 328 patients, both for externally testing the classification model. The datasets were collected from three local institutions from January 2011 to December 2020: dataset 1 was collected from the First Affiliated Hospital of Zhengzhou University (FAHZZU); dataset 2 was collected from Henan Provincial People's Hospital (HPPH); dataset 3 was collected from Xuanwu Hospital Capital Medical University (XHCMU). The inclusion criteria are as follows: (1) adult patients (>18 years) surgically treated and pathologically diagnosed as diffuse gliomas (WHO Grade 2-4), (2) availability of clinical, histological and molecular data, (3) availability of sufficient formalin-fixed, paraffin embedded (FFPE) tumor tissues for testing for molecular markers in the 2021 WHO classification of adult-type diffuse gliomas, (4) availability of H&E slides for scanning as digitalized WSIs, (4) sufficient image quality of digitalized WSIs. The exclusion criteria were incomplete clinical data, or insufficient FFPE tumor tissues for testing for molecular markers, or poor quality of the imaging data. Dataset 1 comprised three cohorts: a (1) training cohort (n = 1362, from FAHZZU) and a (2) validation cohort (n = 340, from FAHZZU) used to develop the classification model, a (3) internal testing cohort (n = 289, from FAHZZU) to validate the model. Dataset 2 was used as an (4) external testing cohort 1 (n = 305, from HPPH) while dataset 3 was used as an (5) external testing cohort 2, both for independently testing the classification model. The selection pipeline for the two datasets and five cohorts was described in Figure 1a. Detailed information of all cohorts is shown in the following.

(1) Training cohort (n = 1362)

In total, 1991 consecutive eligible patients were recruited from FAHZZU. These patients were divided into a training cohort (n = 1362), a validation cohort (n = 340), and an internal testing cohort (n = 289). Patients collected between January 2011 and December 2019 were divided into the training and validation cohorts using stratified random sampling at a ratio of 4:1, with the clinical parameters between both cohorts balanced. This procedure was repeated using a five-fold cross-validation approach, where the patients were divided into a training cohort and a validation cohort five times. Finally, the model with the best performance in classifying the six tumor categories was selected as the optimal model and its performance was further tested in the testing cohorts. Patients collected between January 2020 and December 2020 were used as the internal testing cohort. The training set used to develop the optimal model included 1362 cases (male vs female: 787 vs 575, IDH mutation vs non-mutation: 516 vs 864, mean age: 50.66 years, range: 18 to 86 years).

(2) Validation cohort (n = 340)

The validation cohort including 340 cases (male vs female: 195 vs 145, IDH mutation vs non-mutation: 121 vs 219, mean age: 50.81 years, range: 18 to 78 years) was used for hyperparameter optimization and for internally validating the performance of the finally selected optimal model.

(3) Internal test cohort (n = 289)

This internal cohort including 289 cases (male vs female: 172 vs 117, IDH mutation vs non-mutation: 100 vs 189, mean age: 50.25 years, range: 18 to 77 years) was separately collected from 2020 and was used for further testing the model performance.

(4) External test cohort 1 (n = 305)

The external testing cohort 1 recruited from HPPH included 305 cases (male vs female: 171 vs 134, IDH mutation vs non-mutation: 75 vs 230, mean age: 52.46 years, range: 20 to 77 years). This cohort was used to externally validate the performance of the classification model.

(5) External test cohort 2 (n = 328)

The external testing cohort 2 recruited from XHCMU included 328 cases (male vs female: 186 vs 142, IDH mutation vs non-mutation: 136 vs 192, mean age: 50.83 years, range: 18 to 82 years). This cohort was also used to externally validate the performance of the classification model

For the study cohorts, clinical parameters including gender, age, WHO grade, and subtype were collected. Sanger sequencing or FISH was used for detection of IDH mutation, TERT promoter mutations, 1p/19 co-deletion, CDKN2A/B homozygous deletion, EGFR amplification, and chromosome 7 gain/chromosome 10 loss, as described in Supplementary A2-A3. Based on both histological features and molecular markers, the classification of adult-type diffuse gliomas according to the 2021 WHO rule was made, as described in Supplementary A4.

A2: Detection of IDH mutation and TERT promoter mutations by Sanger sequencing

Mutation analysis of IDH1/IDH2

Mutational hotspots of IDH1/IDH2 were evaluated by direct sequencing. Tissues from representative tumor area (the proportion of tumor cells＞20%) were scrapped off from dewaxed sections and treated with PCR reaction solution A 10μl (reaction mixture containing 1μl of cell lysate, 0.3mM of each dNTP, 2.5mM MgCl2, 0.3μM of each primer and 0.2U of KAPA HiFi HotStart DNA Polymerase (Kapa Biosystems Inc., Wilmington, USA)), Shrimp Alkaline Phosphatase (SAP) enzyme (NEB, Ipswich, MA, USA) 2μl and BigDye (BigDye™ Terminator v3.1 Cycle Sequencing Kit, Thermo Fisher Scientific, Waltham, MA, USA) 1μl for centrifugation at 1600g rpm for 10 sec. The crude cell lysate was centrifuged and supernatant was used for subsequent PCR analysis. The forward primer primers (IDH1-F:5’-CGGTCTTCAGAGAAGCCATT-3’,IDH1-R:5’-CACATTATTGCCAACATGAC

-3’,IDH2-F:5’-AGCCCATCATCTGCAAAAAC-3’,IDH2-R:5’-CTAGGCGAGGAGCTCCAGT-3’) were used to amplify the region of mutational hotspots of IDH1/IDH2. ①PCR was performed was initiated at 95°C for 5 min, followed by 40 cycles of 95°C for 20 sec, 57°C for 30 sec and 72°C for 1min, and a final extension of 72°C for 5 min and 10°C for 10 min. ② 5μl PCR products were then mixed with 2μl SAP enzyme and reacted at 37°C for 40min and then at 80° C for 15min. ③Then 18μl PCR reaction solution C（CWBIO, Beijing, Chima）, 1μl products from ② step, and 1μl BigDye were mixed and reacted at 96°C for 1 min, followed by 30 cycles of 96°C for 10 sec, 50° C for 5 sec and 60° C for 2 min, and a final extension of 25°C for 1 min and 10°C for 10 min. Then 50μl natrium asceticism-ethanol mixture (3M NaAc: ethanol=1:15) were added and the mixture was centrifuged for 30min (12000 rpm, 4°C), with the supernatant being discarded. Then 70μl 75% ethanol were added and the mixture was centrifugated for 15min (12000 rpm, 4°C), with the supernatant being discarded. After complete volatilization of the ethanol at room temperature, 12μl Hi-Di™ Formamide (Thermo Fisher Scientific, Waltham, MA, USA) were added into the precipitate to dissolve the DNA. The dissolved products were sequenced on Applied Biosystems™ 3500DxGenetic Analyzer (Thermo Fisher Scientific, Waltham, MA, USA), and analyzed by Chromas software (Technelysium, South Brisbane, Australia). The sequencing results were compared with wild-type sequences of IDH1/IDH2 for analysis.

Mutation Analysis of TERT promoter

Tissue samples were prepared according to the “Mutation Analysis of IDH1/IDH2 protocol” protocol previous described. The crude cell lysate was centrifuged, and supernatant was used for subsequent PCR analysis. The forward primer TERT-F (5’-GTCCTGCCCCTTCACCTT-3’) and reverse primer TERT-R (5’-CAGCGCTGCCTGAAACTC-3’) were used to amplify a 163bp fragment spanning the two mutational hotspots [chr5, 1,295,228 (C228T) and 1,295,250 (C250T)] in TERT promoter region. ①PCR was performed was initiated at 95°C for 5 min, followed by 40 cycles of 95°C for 20 sec, 57°C for 30 sec and 72°C for 1min, and a final extension of 72°C for 5 min and 10°C for 10 min. ②5μl PCR products were then mixed with SAP enzyme and reacted at 37°C for 40 min and then at 80° C for 15 min. ③Then 18μl PCR reaction solution C, 1μl products from ② step, and 1μl BigDye were mixed and reacted at 96°C for 1 min, followed by 30 cycles of 96°C for 10 sec, 50° C for 5 sec and 60° C for 2min, and a final extension of 25°C for 1 min and 10°C for 10 min. The following steps were performed according to the “Mutation Analysis of IDH1/IDH2 protocol” protocol previous described. The sequencing results were compared with wild-type sequences of TERT for analysis.

A3: Detection of chromosome 1p/19q, CDKN2A, EGFR, and chromosome 7/10 status by Fluorescence in Situ Hybridization (FISH)

Chromosome 1p/19q, CDKN2A (9p21) gene, EGFR amplification, and chromosome 7

gain/chromosome 10 loss status was examined by fluorescence in situ hybridization. 4μm thick FFPE sections were baked at 65°C for 2-3h and deparaffinized in xylene for 10 minutes for 2 times. The sections were hydrated by 100％ethanol for 2 min, 85％ethanol for 2 min and 70％ethanol for 2 min orderly, and then immerged in deionized water for 3 min. The sections were processed with citrate repair solutionin (pH6.0) for 4 min in high pressure condition, and then rinsed in 2×SSC solution for 5 min for 2 times. The sections were immerged in protease K fluid（200μg/ml）and incubated for 2 min at 37°C, and then rinsed in 2×SSC solution for 5 min for 2 times. 10μl probes (GP Medical Technologies, Beijing, China) mixture was added to thehybridization zone of the section, and the denaturation and hybridization process was carried out on the ThermoBrite®hybridization instrument (Leica Biosystems, Nussloch, Germany), with denaturation temperature at 83°C for 5 min and hybridization temperature at 42°C for 16h. Sections were immerged in 0.4×SSC plus 0.3% NP-40 cleaning solution (65±1°C) and vibrated for 3 sec. Sections were then retrieved 2 min later and put into 0.1% NP-40 plus 2×SSC cleaning solution at room temperature, vibrated for 3 sec and cleansed for 1 min. Then the sections were immerged in 70% ethanol for 3 min and dried avoiding light at room temperature. 15μl DAPI redyeing agent was added into the hybridization zone of the section, and the section was placed avoiding light for 10 min. At last, the section was placed under the BX51TRF fluorescence microscope (Olympus, Tokyo, Japan) for analysis by expert pathologist (Dr. Wei-wei Wang). Hybridizing signals in at least 100 non-overlapping nuclei were counted.

Chromosome 1p/19q status: The loci interrogated were 1p36.3 (RP11-62M23 labeled red)/1q25.3-q31.1 (RP11-162L13 labeled green) and 19q13.3 (CTD-2571L23 labeled red)/19p12 (RP11-420K14 labeled green). A sample was considered 1p or 19q deleted according to the ratio of number of red signal to green signal. In 1p36 or 19q13, positive loss of heterozygosity (LOH) was determined when the ratio of number of red signal to green signal was less than 0.7.

CDKN2A (9p21) gene status: CDKN2A (9p21) is labeled red and centromere of Chromosome 9 (CSP9) is labeled green. A sample was considered CDKN2A homozygous deficiency according to red signal lost in more than 25 nuclei, at least 100 non-overlapping nuclei were counted. The probes used for FISH were from LBP Medicine Science & Technology Co., Ltd. (Guangzhou, China).

EGFR amplification: EGFR gene is labeled red, and centromere of Chromosome 7 (CSP7) is labeled green. A sample was considered EGFR amplification when the ratio of number of red signal to green signal was greater than 2.

Chromosome 7 gain/chromosome 10 loss status: chromosome 7 and chromosome 10 are both labeled green. A sample was considered chromosome 7 gain and chromosome 10 loss when the number of chromosome 7 signal was greater than 2 and the number of chromosome 10 signal was less than 2.

A4: 2021 WHO Classification of adult-type diffuse gliomas in our datasets

According to 2021 WHO CNS5, the classification of adult-type diffuse gliomas in our study was showed as following: IDH1 and IDH2 mutation were detected by Sanger sequencing firstly. For the cases having IDH1 or IDH2 mutation, ATRX expression were determined by immunohistochemistry (IHC)(1:1, ZA-0016, ZSGB-BIO, China), when the results of ATRX IHC was negative, the cases in the presence of typical necrosis and/or microvascular proliferation (MVP) were classified as astrocytoma, IDH-mutant, Grade 4 (A4); For the cases without necrosis or MVP, CDKN2A gene status was then detected by FISH, and the cases were also classified as astrocytoma, IDH-mutant, Grade 4 (A4) if CDKN2A gene was homozygous deficiency, otherwise the cases were classified as astrocytoma, IDH-mutant, Grade 2 (A2) which having nil or low mitosis (< 2 per case) or were classified as astrocytoma, IDH-mutant, Grade 3 (A3) which having high mitosis (>= 2 per case). When the results of ATRX IHC was positive, chromosome 1p/19q status were detected by FISH, if 1p/19q were co-deletion, the cases were classified as Oligodendrolioma, IDH-mutant, Grade 2/3, which were further classified as Oligodendrolioma, IDH-mutant, Grade 2 (O2) having nil or low mitosis (< 2 per case), or classified as Oligodendrolioma, IDH-mutant, Grade 3 (O3) having high mitosis (>= 2 per case); If 1p/19q were none co-deletion, the cases were classified as astrocytoma, IDH-mutant and the classification was done through the workflow above. Glioblastoma, IDH-wild type (GBM) has been diagnosed in the setting of IDH-wild type diffuse astrocytic glioma in adults if there existed typical necrosis and/or MVP, or TERT promoter mutations, or EGFR amplification, or Chromosome 7 gain/chromosome 10 loss (+ 7/− 10).

A5: The patch clustering algorithm

We aimed to select a subset of patches within a WSI to reduce the computational burden. Specifically, we extracted many patches from a WSI and then partitioned these patches into several clusters according to their "phenotypes", or important cancerous features that were useful in subtype/grade discrimination. Considering the less representative power of features from original images, we extracted deep features using convolutional neural networks (CNNs) for patch clustering. Here a ResNet-50 network pretrained with patch-level labels on all patches in the training cohort was used as the deep feature extractor. Having this pretrained ResNet-50, a patch can be fed into it and 2048 features can be extracted from its average pooling layer. As this ResNet-50 was trained for patch-level tumor classification into six categories (A2, A3, A4, O2, O3, and GBM), the features extracted using this network were used as cancerous phenotypes relevant to tumor subtypes. Next, we randomly selected 100 patients in the training cohort, including 11 A2, 2 A3, 2 A4, 14 O2 3 O3, and 68 GBM patients, for training a deep feature-based K-means algorithm for patch clustering. From these

100 patients, 43653 patches were extracted and were fed into the pretrained ResNet-50 for deep feature extraction. From each of the 42216 patches, 2046 deep features were extracted. Using these 43653×2046=89314038 features, the candidate 42653 patches were used to develop a K-means clustering algorithm by partitioning these patches into K clusters. To improve the clustering stability, K-means++ approach with multiple initializations was used. The K-means clustering was performed by using a KMeans function in the scikit-learn python library, with the function parameters init = 'k-means++' and n_init = 10. Here the optimal cluster number K=9 was determined when the silhouette coefficient reached its maximum value, as shown in Figure 3. We also calculated another metrics named Calinski-Harabasz index to assess the clustering quality. At K=9 the Calinski-Harabasz index also reached its highest point, providing additional support for this optimal cluster number. The patches belonged to different clusters were considered to have different discriminative powers as they may have distinguished imaging patterns relevant to tumor types/grades.

A6: Deep learning network architecture and training scheme

There were three kinds of CNNs: the CNN used as deep feature extractor in patch clustering step, the CNNs used for cluster-based tumor classification in patch selecting step, and the CNN used for patch-level tumor classification. All the CNNs employed the same ResNet-50 architecture, as shown in the following Supplementary Table 4.

Supplementary Table 4 Network architecture and parameters.

**Network layers**	**output size**	**parameters**
conv1	112×112	7×7, 64, stride=2
Max pooling	56×56	3×3, stride=2
Residual block1	56×56	$\begin{bmatrix} 1\times1, & 64 \\ 3\times3, & 64 \\ 1\times1, & 256 \end{bmatrix} \times 3$
Residual block2	28×28	$\begin{bmatrix} 1\times1, & 128 \\ 3\times3, & 128 \\ 1\times1, & 512 \end{bmatrix} \times 4$
Residual block3	14×14	$\begin{bmatrix} 1\times1, & 256 \\ 3\times3, & 256 \\ 1\times1, & 1024 \end{bmatrix} \times 6$
Residual block4	7×7	$\begin{bmatrix} 1\times1, & 512 \\ 3\times3, & 512 \\ 1\times1, & 2048 \end{bmatrix} \times 3$
Average pooling	1×2048	7×7, stride=1
Full connected layer	512	512
Output layer	6	6

The network inputs were small patches extracted from the whole slide image. The patch-level labels were used in all CNNs. For the CNN feature extractor, namely the all-patch

classifier, all patches in the training cohort were used to train the network. There were nine cluster-based CNN classifiers, corresponding to the nine patch clusters. Each of them was trained using one patch cluster in the training cohort. The patch-level CNN was trained using the patches from the three selected clusters in the training cohort. All CNNs were trained from scratch while patches in the validation set were used for hyperparameter optimization and for optimal model selection. In the cross-validation process, the model was trained for a minimum of 50 epochs. Then, the loss on the validation set was computed in each epoch, and the model with the lowest average validation loss over 10 consecutive epochs was saved. If such a model was not found, the training continued up to a maximum of 150 epochs and the last model was saved. During training, an SGD optimizer was used with a learning rate of 0.001 and a batch size of 64. To accommodate the class imbalance problem caused by the different incidence rate across the glioma types, we used random rotation and shear approaches to augment A3, A4, and O3 classes in the training dataset (after training/validation dataset division). The sample number in each of the three classes have tripled after augmentation. The output of the network was the probability values of each glioma subtype. The loss function for this CNN training was the cross-entropy loss function as

$$\mathrm{loss} = -\sum_{i=1}^{n}[p(x_i) \times \log(q(x_i))]$$

where i was the index of patient; $p(x_i)$ was the true probability value of patient i, i.e., the label of input data; $q(x_i)$ was the predicted probability value of patient i, i.e., the output of the network. The network was implemented using the PyTorch (version 1.4.0) library. The same training parameters were used across each fold in the cross-validation procedure. Finally, the patient-level classifier with the highest mean AUC over the six types (A2, A3, A4, O2, O3 and GBM) on the validation cohort was selected as the optimal model and its performance was further tested on the testing cohorts.

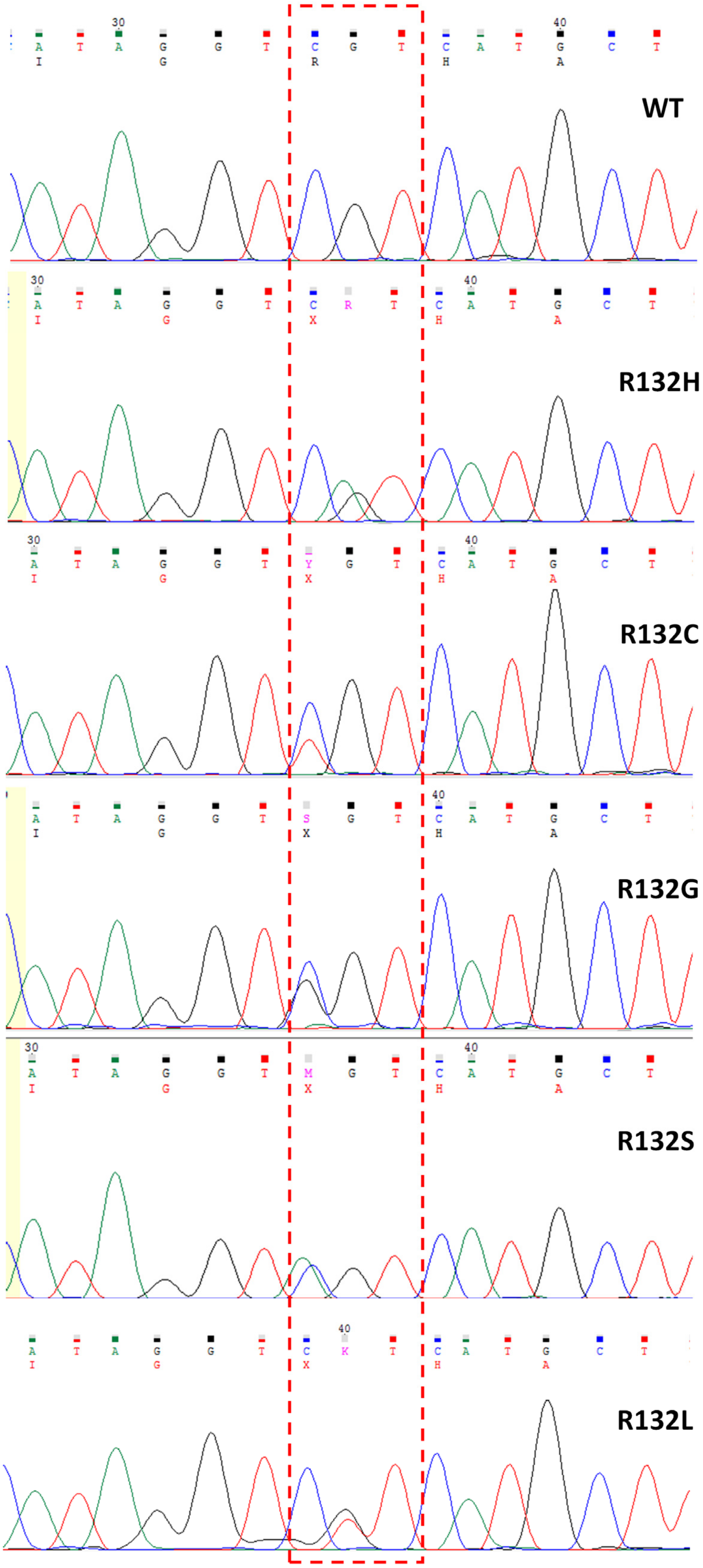

Supplementary Figure 1 Representative results of IDH1 mutation. Mutational hotspots of IDH1 at codon 132H, 132C, 132G, 132S, 132L were evaluated by Sanger sequencing.

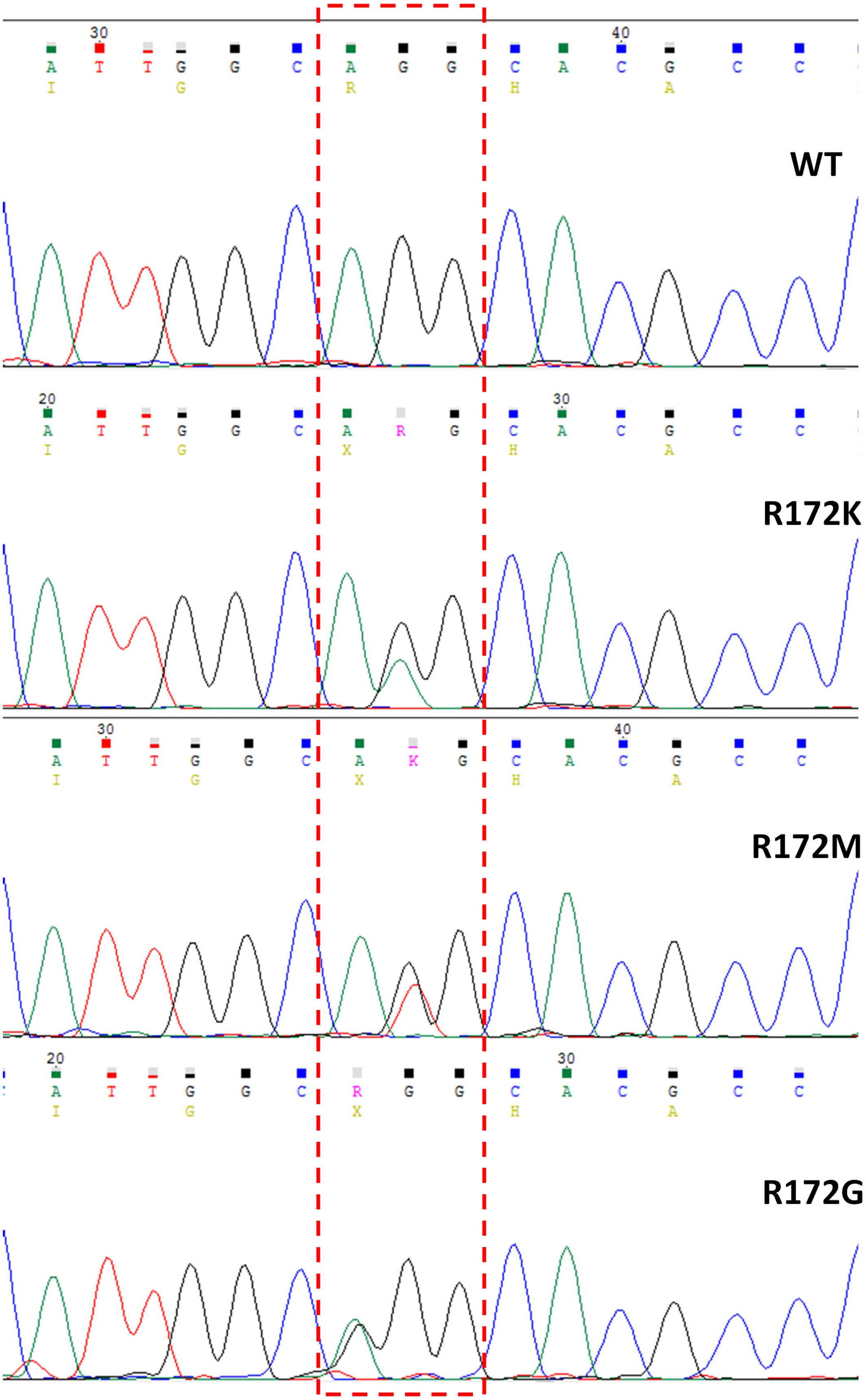

Supplementary Figure 2 Representative results of IDH2 mutation. Mutational hotspots of IDH2 at codon 172K, 172M, 172G were evaluated by Sanger sequencing.

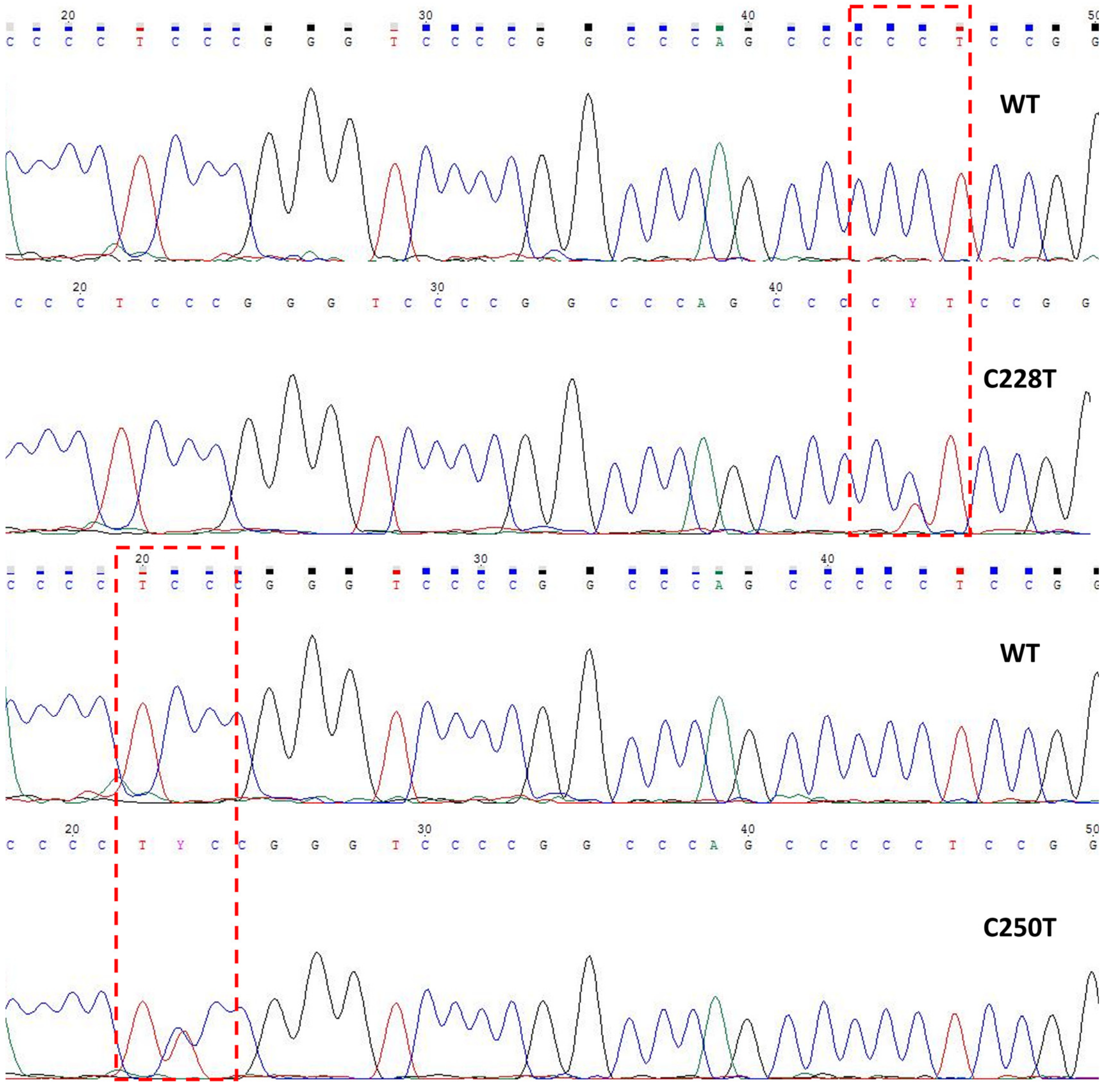

Supplementary Figure 3 Representative results of TERT promoter mutation. Mutational hotspots of TERT promoter at codon C228T and C250T were evaluated by Sanger sequencing.

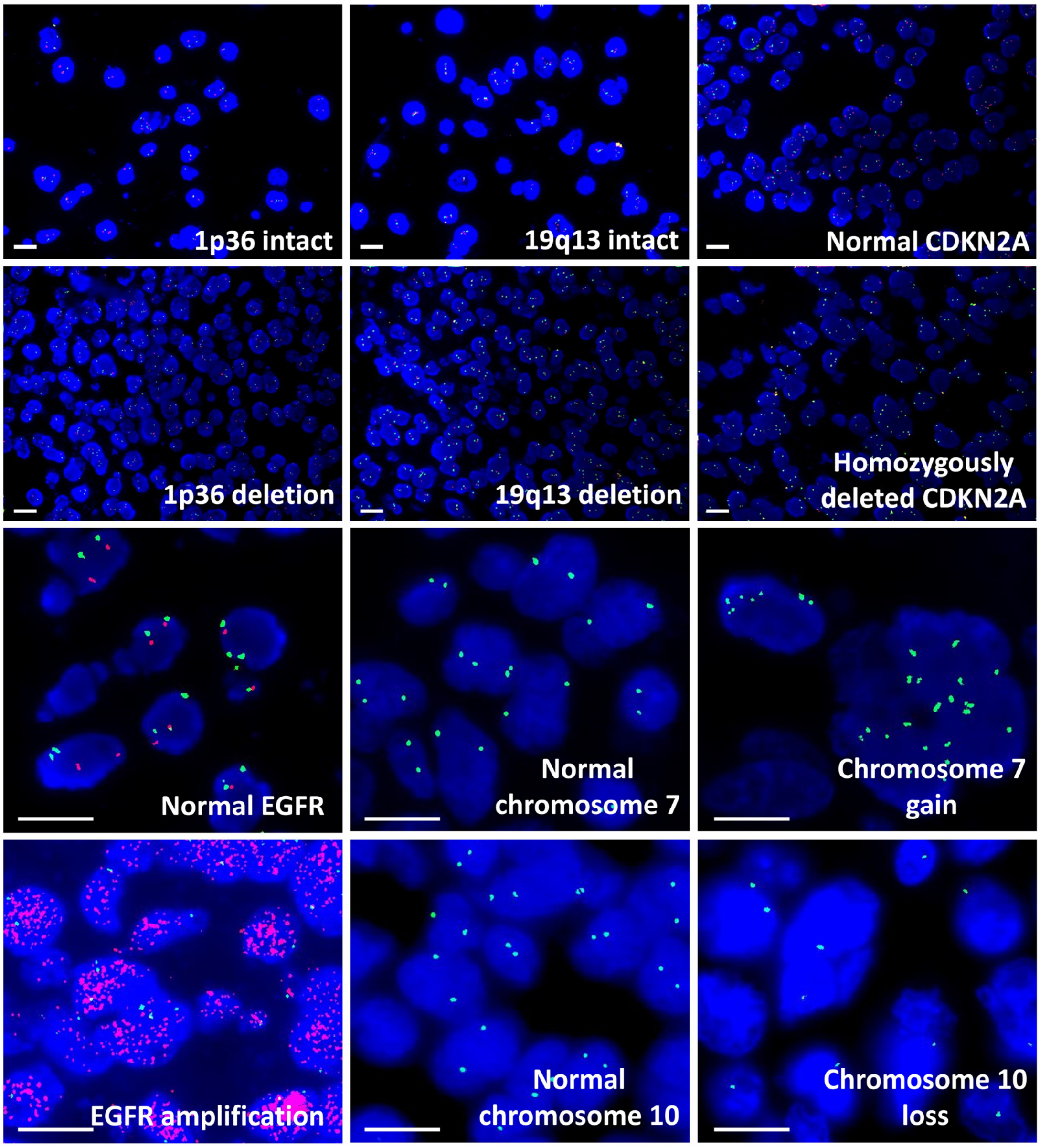

Supplementary Figure 4 Representative results of 1p/19q deletions, CDKN2A homozygous deletion, EGFR amplification and chromosome 7 gain/chromosome 10 loss. Chromosome 1p/19q deletions, CDKN2A homozygous deletion, EGFR amplification and chromosome 7 gain/chromosome 10 loss were evaluated by fluorescence in situ hybridization (FISH). Scale bars, 25μm. All experiments were performed at least three times independently.

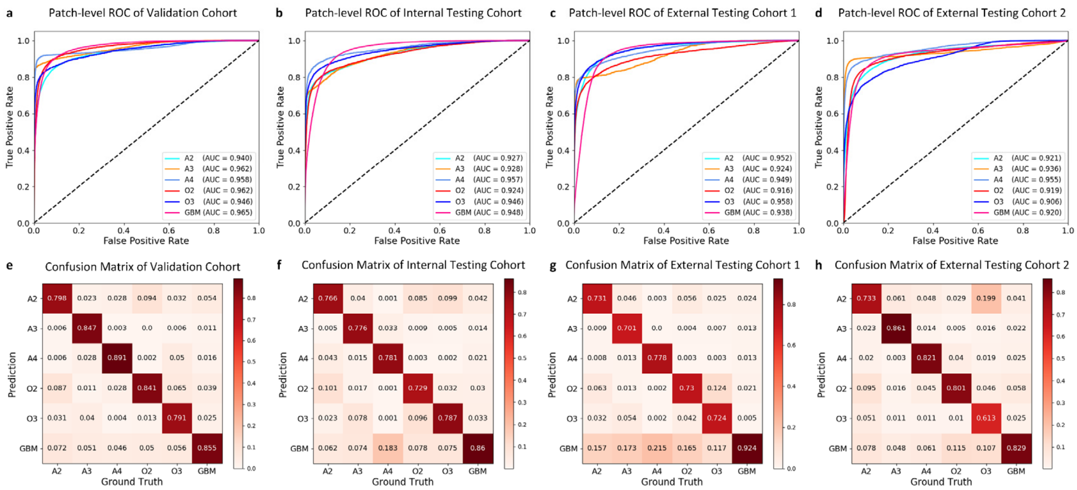

Supplementary Figure 5 The patch-level classification performance of the presented clustering-based diagnostic model in classifying the six categories of A2,3,4, O2,3, and GBM. (a-d): ROC curves of the patch-level classifier trained using the patches of the three selected clusters in the internal validation cohort (a), internal testing cohort (b), external testing cohort 1 (c), and external testing cohort 2 (d), respectively. (e-h): confusion matrix of the patch-level classifier in the internal validation cohort (e), internal testing cohort (f), external testing cohort 1 (g), and external testing cohort 2 (h), respectively. Source data are provided as a Source Data file.

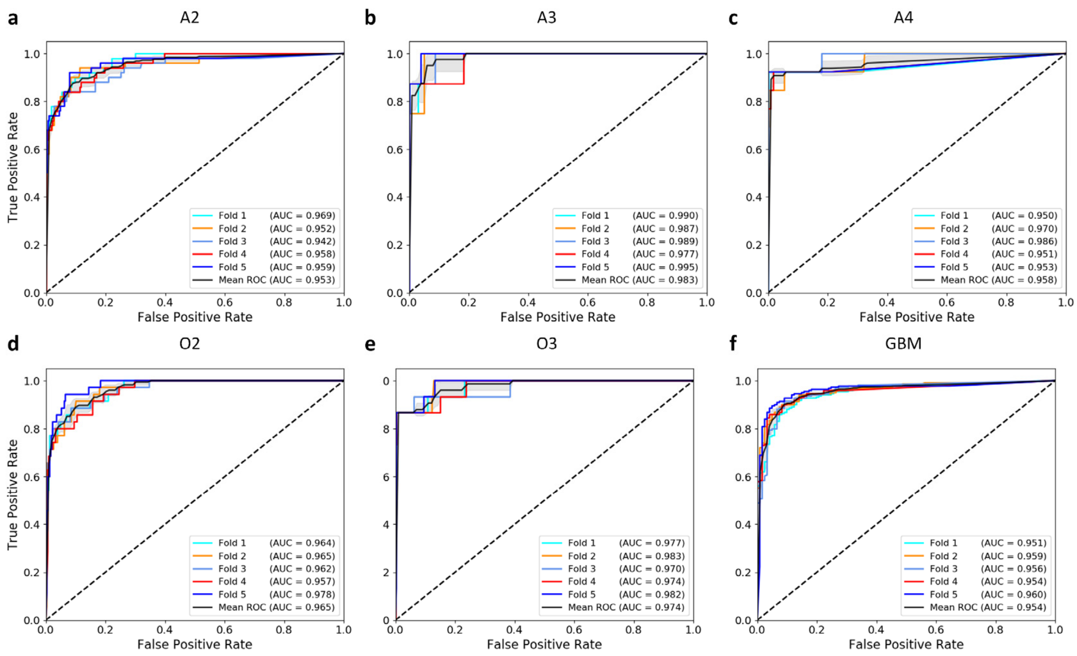

Supplementary Figure 6 The ROC curves for classifying the six categories A2 (a), A3 (b), A4 (c), O2 (d), O3 (e), and GBM (f) on the validation cohort in the five-fold cross-validation. Patients from FAHZZU collected from January 2011 to December 2019 were divided into a training cohort (n = 1362) and a validation cohort (n = 340) with stratified random sampling at a ratio of 4:1, with the clinical parameters balanced. We repeated this procedure in a five-fold cross-validation, re-assigning the patients into training and validation cohorts five times. The ROC curves for classifying the six categories on the validation cohort in each fold was plotted in subfigure (a-f), respectively, where the mean ROC over all folds for each category was also plotted. The gray area between the bounds defined by the mean true positive rates (TPR) ± standard deviation across all folds visually highlighted the range of variability of the model performance. A narrower gray region indicated more reliability in the model prediction. Finally, the fifth model with the highest average AUC of 0.971 over all six categories was selected as the optimal model, corresponding to the ROC curves for fold 5. A2: Astrocytoma, IDH-mutant, Grade 2; A3: Astrocytoma, IDH-mutant, Grade 3; A4: Astrocytoma, IDH-mutant, Grade 4; O2: IDH-mutant, and 1p/19q-codeleted, Grade 2; O3: IDH-mutant, and 1p/19q-codeleted, Grade 3; GBM: Glioblastoma, IDH-wildtype, Grade 4. Source data are provided as a Source Data file.

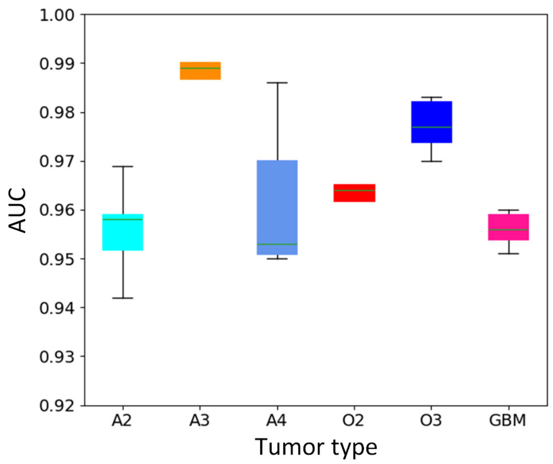

Supplementary Figure 7 The boxplots of the AUCs of the models obtained in five-fold cross-validation for the six categories of A2, A3, A4, O2, O3, and GBM in the validation cohort. In cross-validation, patients from FAHZZU from January 2011 to December 2019 were randomly divided into non-overlapping training (n = 1362) and validation (n = 340) cohorts five times at a ratio of 4:1, generating five models. Boxplots summarized the AUCs of the five models in classifying the six categories in the validation cohort. For all boxplots, center line inside the box represents the median, or the 50th percentile; upper and lower bounds of the box represent the 75th and 25th percentiles, respectively; whiskers extend from the box to the minimum and maximum values that are within 1.5 times the interquartile range from the box bounds, where the interquartile range equals the distance between the upper and lower box bounds. A2: Astrocytoma, IDH-mutant, Grade 2; A3: Astrocytoma, IDH-mutant, Grade 3; A4: Astrocytoma, IDH-mutant, Grade 4; O2: IDH-mutant, and 1p/19q-codeleted, Grade 2; O3: IDH-mutant, and 1p/19q-codeleted, Grade 3; GBM: Glioblastoma, IDH-wildtype, Grade 4. Source data are provided as a Source Data file.

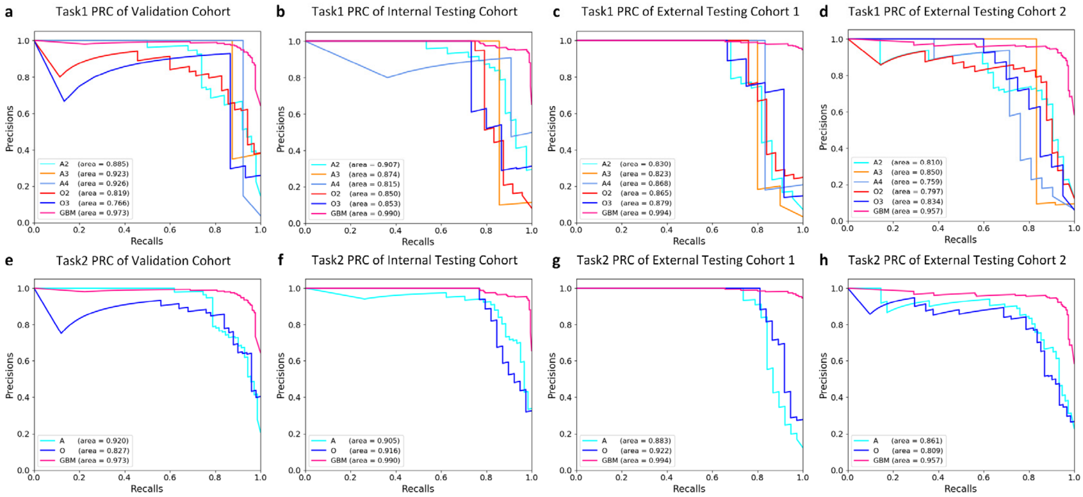

Supplementary Figure 8 The patient-level classification performance of the presented clustering-based diagnostic model on task 1-2. (a-d, task 1): PR curves for classifying the six categories of A2,3,4, O2,3, and GBM on the internal validation cohort (a), internal testing cohort (b), external testing cohort 1 (c), and external testing cohort 2 (d), respectively. (e-h, task 2): PR curves for classifying the three major types of A, O, and GBM on the internal validation cohort (e), internal testing cohort (f), external testing cohort 1 (g), and external testing cohort 2 (h), respectively. A2: Astrocytoma, IDH-mutant, Grade 2; A3: Astrocytoma, IDH-mutant, Grade 3; A4: Astrocytoma, IDH-mutant, Grade 4; O2: IDH-mutant, and 1p/19q-codeleted, Grade 2; O3: IDH-mutant, and 1p/19q-codeleted, Grade 3; GBM: Glioblastoma, IDH-wildtype, Grade 4. Source data are provided as a Source Data file.

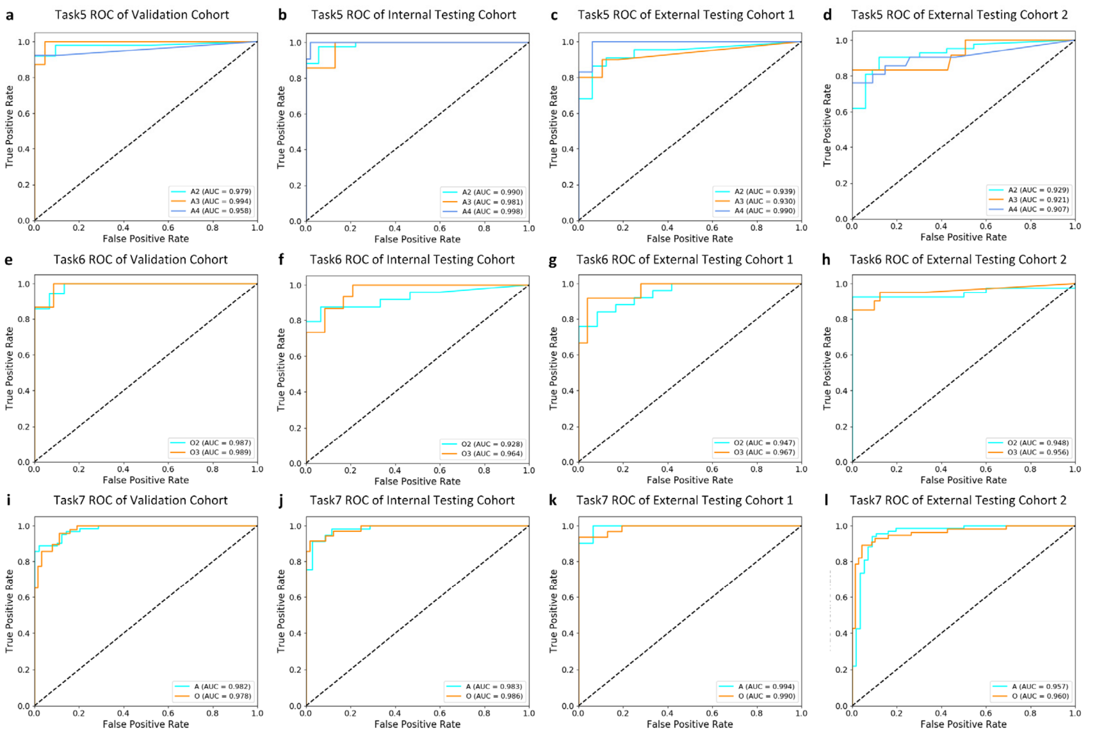

Supplementary Figure 9 The patient-level classification performance of the presented clustering-based diagnostic model on task 5-7. (a-d, task 5): ROC curves for classifying A2, A3, and A4 within the IDH-mutant astrocytoma type on the internal validation cohort (a), internal testing cohort (b), external testing cohort 1 (c), and external testing cohort 2 (d), respectively. (e-h, task 6): ROC curves for classifying O2 and O3 within the oligodendroglioma type on the internal validation cohort (e), internal testing cohort (f), external testing cohort 1 (g), and external testing cohort 2 (h), respectively. (i-l, task 7): ROC curves for classifying IDH-mutant astrocytoma and IDH-mutant 1p/19q-codeleted oligodendroglioma on the internal validation cohort (i), internal testing cohort (j), external testing cohort 1 (k), and external testing cohort 2 (l), respectively. Corresponding classification results can be found in Table 1. A2: Astrocytoma, IDH-mutant, Grade 2; A3: Astrocytoma, IDH-mutant, Grade 3; A4: Astrocytoma, IDH-mutant, Grade 4; O2: IDH-mutant, and 1p/19q-codeleted, Grade 2; O3: IDH-mutant, and 1p/19q-codeleted, Grade 3; GBM: Glioblastoma, IDH-wildtype, Grade 4. Source data are provided as a Source Data file.

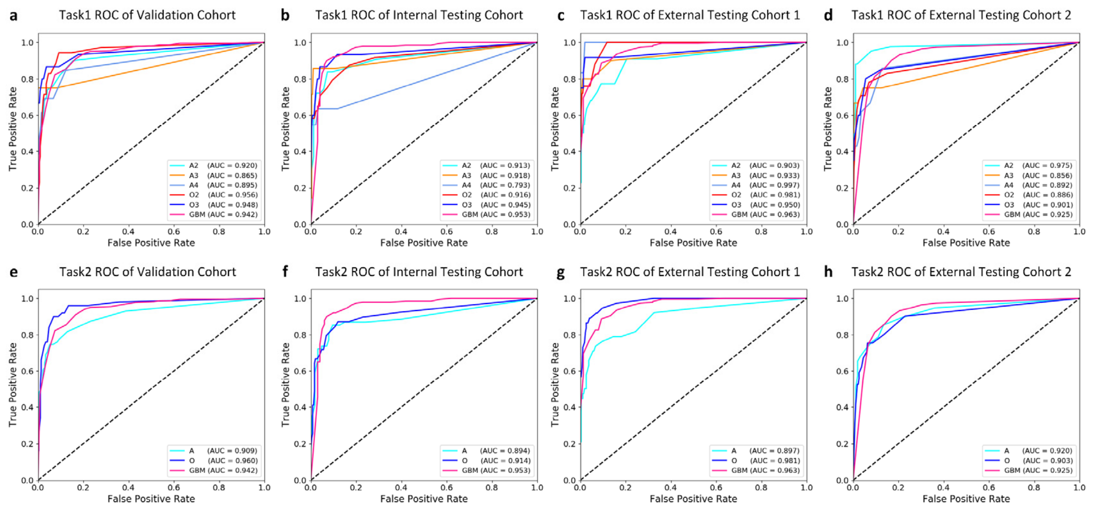

Supplementary Figure 10 The patient-level classification performance of the traditional MIL model on task 1-2. (a-d, task 1): ROC curves for classifying the six categories of A2,3,4, O2,3, and GBM on the internal validation cohort (a), internal testing cohort (b), external testing cohort 1 (c), and external testing cohort 2 (d), respectively. (e-h, task 2): ROC curves for classifying the three major types of A, O, and GBM on the internal validation cohort (e), internal testing cohort (f), external testing cohort 1 (g), and external testing cohort 2 (h), respectively. Corresponding classification results can be found in Supplementary Data 1. A2: Astrocytoma, IDH-mutant, Grade 2; A3: Astrocytoma, IDH-mutant, Grade 3; A4: Astrocytoma, IDH-mutant, Grade 4; O2: IDH-mutant, and 1p/19q-codeleted, Grade 2; O3: IDH-mutant, and 1p/19q-codeleted, Grade 3; GBM: Glioblastoma, IDH-wildtype, Grade 4. Source data are provided as a Source Data file.

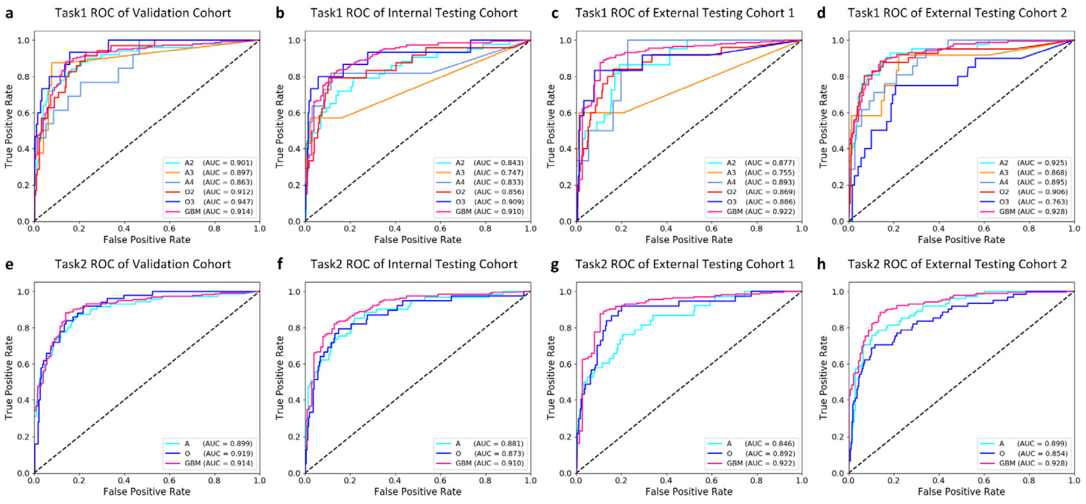

Supplementary Figure 11 The patient-level classification performance of the all-patch model on task 1-2. (a-d, task 1): ROC curves for classifying the six categories of A2,3,4, O2,3, and GBM on the internal validation cohort (a), internal testing cohort (b), external testing cohort 1 (c), and external testing cohort 2 (f), respectively. (e-h, task 2): ROC curves for classifying the three major types of A, O, and GBM on the internal validation cohort (e), internal testing cohort (f), external testing cohort 1 (g), and external testing cohort 2 (h), respectively. Corresponding classification results can be found in Supplementary Data 2. A2: Astrocytoma, IDH-mutant, Grade 2; A3: Astrocytoma, IDH-mutant, Grade 3; A4: Astrocytoma, IDH-mutant, Grade 4; O2: IDH-mutant, and 1p/19q-codeleted, Grade 2; O3: IDH-mutant, and 1p/19q-codeleted, Grade 3; GBM: Glioblastoma, IDH-wildtype, Grade 4. Source data are provided as a Source Data file.

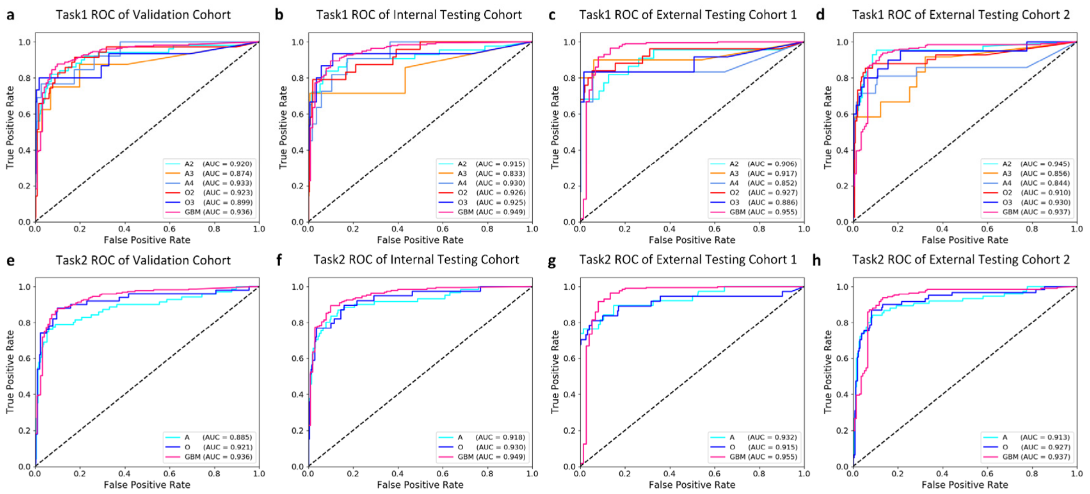

Supplementary Figure 12 The patient-level classification performance of the attention-based MIL (AMIL) model on task 1-2. (a-d, task 1): ROC curves for classifying the six categories of A2,3,4, O2,3, and GBM on the internal validation cohort (a), internal testing cohort (b), and external testing cohort 1 (c), and external testing cohort 2 (d), respectively. (e-h, task 2): ROC curves for classifying the three major types of A, O, and GBM on the internal validation cohort (e), internal testing cohort (f), external testing cohort 1 (g), and external testing cohort 2 (h), respectively. Corresponding classification results can be found in Supplementary Table 2. A2: Astrocytoma, IDH-mutant, Grade 2; A3: Astrocytoma, IDH-mutant, Grade 3; A4: Astrocytoma, IDH-mutant, Grade 4; O2: IDH-mutant, and 1p/19q-codeleted, Grade 2; O3: IDH-mutant, and 1p/19q-codeleted, Grade 3; GBM: Glioblastoma, IDH-wildtype, Grade 4. MIL: multiple-instance learning. Source data are provided as a Source Data file.

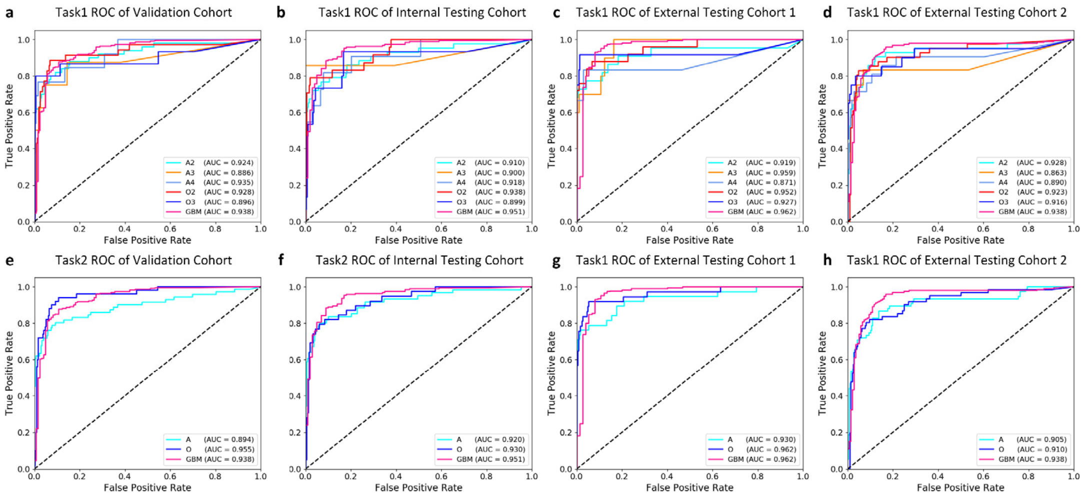

Supplementary Figure 13 The patient-level classification performance of the clustering-constraint-attention MIL (CLAM) model on task 1-2. (a-d, task 1): ROC curves for classifying the six categories of A2,3,4, O2,3, and GBM on the internal validation cohort (a), internal testing cohort (b), and external testing cohort 1 (c), and external testing cohort 2 (d), respectively. (e-h, task 2): ROC curves for classifying the three major types of A, O, and GBM on the internal validation cohort (e), internal testing cohort (f), external testing cohort 1 (g), and external testing cohort 2 (h), respectively. Corresponding classification results can be found in Supplementary Table 2. A2: Astrocytoma, IDH-mutant, Grade 2; A3: Astrocytoma, IDH-mutant, Grade 3; A4: Astrocytoma, IDH-mutant, Grade 4; O2: IDH-mutant, and 1p/19q-codeleted, Grade 2; O3: IDH-mutant, and 1p/19q-codeleted, Grade 3; GBM: Glioblastoma, IDH-wildtype, Grade 4. MIL: multiple-instance learning. Source data are provided as a Source Data file.

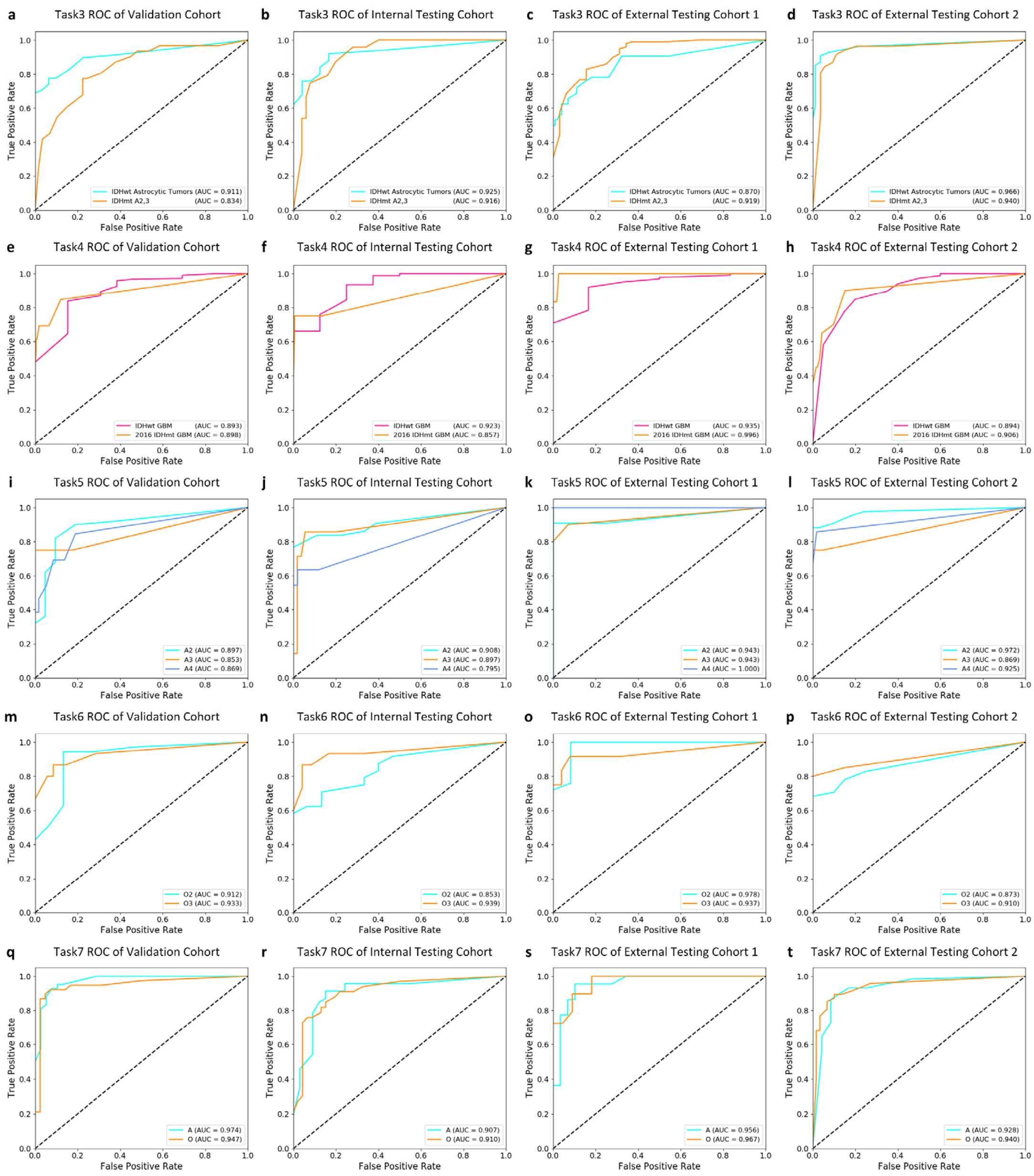

Supplementary Figure 14 The patient-level classification performance of the traditional MIL model on task 3-7. (a-d, task 3): ROC curves for distinguishing the two subgroups of IDH-mutant astrocytic tumors A2-3 and IDH-wildtype diffuse astrocytic tumors without the histological features of glioblastoma (classified as glioblastoma). (e-h, task 4): ROC curves for distinguishing the two subgroups of IDH-mutant astrocytic tumors A2 and IDH-wildtype gliomas. (i-l, task 5): ROC curves for classifying A2, A3, and A4 within the IDH-mutant astrocytoma subgroup. (m-p, task 6): ROC curves for classifying O2 and O3 within the oligodendroglioma subgroup. (q-t, task 7): ROC curves for classifying IDH-mutant astrocytoma and IDH-mutant 1p/19q-codeleted oligodendroglioma. Corresponding classification results can be found in Supplementary Data 1. A2: Astrocytoma, IDH-mutant, Grade 2; A3: Astrocytoma, IDH-mutant, Grade 3; A4: Astrocytoma, IDH-mutant, Grade 4; O2: IDH-mutant, and 1p/19q-codeleted, Grade 2; O3: IDH-mutant, and 1p/19q-codeleted,

Grade 3; GBM: Glioblastoma, IDH-wildtype, Grade 4. MIL: multiple-instance learning. Source data are provided as a Source Data file.

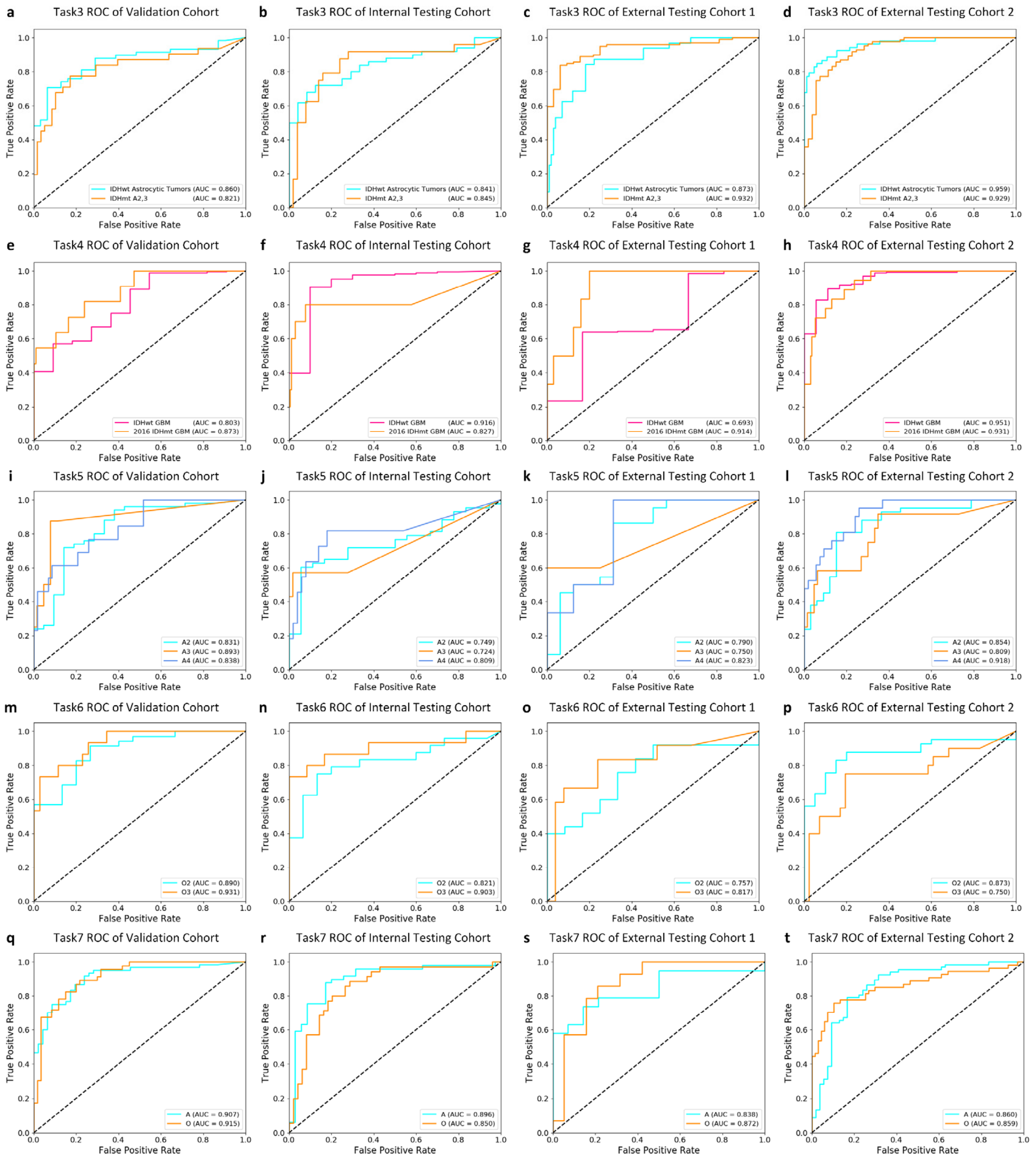

Supplementary Figure 15 The patient-level classification performance of the all-patch model on task 3-7. (a-d, task 3): ROC curves for distinguishing the two subgroups of IDH-mutant astrocytic tumors A2-3 and IDH-wildtype diffuse astrocytic tumors without the histological features of glioblastoma (classified as glioblastoma). (e-h, task 4): ROC curves for distinguishing the two subgroups of IDH-mutant astrocytic tumors A2 and IDH-wildtype gliomas. (i-l, task 5): ROC curves for classifying A2, A3, and A4 within the IDH-mutant astrocytoma subgroup. (m-p, task 6): ROC curves for classifying O2 and O3 within the oligodendroglioma subgroup. (q-t, task 7): ROC curves for classifying IDH-mutant astrocytoma and IDH-mutant 1p/19q-codeleted oligodendroglioma. Corresponding classification results can be found in Supplementary Data 2. A2: Astrocytoma, IDH-mutant, Grade 2; A3: Astrocytoma, IDH-mutant, Grade 3; A4: Astrocytoma, IDH-mutant, Grade 4; O2: IDH-mutant, and 1p/19q-codeleted, Grade 2; O3: IDH-mutant, and 1p/19q-codeleted,

Grade 3; GBM: Glioblastoma, IDH-wildtype, Grade 4. Source data are provided as a Source Data file.

Supplementary Table 1 Characteristics of patients and tumors in all datasets.

Characteristic	Training cohort (n=1362)	Validation cohort (n=340)	*P* values	Internal testing cohort (n=289)	*P* values	External testing cohort 1 (n=305)	*P* values	External testing cohort 2 (n = 328)	*P* values
**Sex**			0.89		0.59		0.58		0.36
**Male**	787 (58%)	195 (57%)		172 (60%)		171 (56%)		186 (57%)	
**Female**	575(42%)	145 (43%)		117 (40%)		134 (44%)		142 (43%)	
**Age(years)**	50.66±12.91	50.81±12.33	0.19	50.25±13.08	0.62	52.46±12.82	0.03	50.82±14.25	0.27
**IDH mutations**			0.74		0.52		6.7e-5		0.05
**Yes**	516 (38%)	121 (36%)		100 (35%)		75 (25%)		136 (41%)	
**No**	864 (63%)	219 (64%)		189 (65%)		230 (75%)		192 (59%)	
**Subtypes/Grades**			0.13		0.08		0.06		0.09
**A2**	191 (14%)	50 (15%)		43 (15%)		22 (7%)		42 (13%)	
**A3**	30 (2%)	8 (2%)		7 (2%)		10 (3%)		12 (4%)	
**A4**	37 (3%)	13 (4%)		11 (4%)		6 (2%)		21 (6%)	
**O2**	171 (13%)	35 (10%)		24 (8%)		25 (8%)		41 (13%)	
**O3**	87 (6%)	15 (4%)		15 (5%)		12 (4%)		20 (6%)	
**GBM**	846 (62%)	219 (64%)		189 (65%)		230 (75%)		192 (59%)	

A2: Astrocytoma, IDH-mutant, Grade 2; A3: Astrocytoma, IDH-mutant, Grade 3; A4: Astrocytoma, IDH-mutant, Grade 4; O2: Oligodendroglioma, IDH-mutant, and 1p/19q-codeleted, Grade 2; O3: Oligodendroglioma, IDH-mutant, and 1p/19q-codeleted, Grade 3; GBM: Glioblastoma, IDH-wildtype, Grade 4. *P* values were calculated using the two-sided Wilcoxon test for age as a continuous variable or Chi-square test for categorical variables.

Supplementary Table 2 A summary of the comparison of model performance in terms of AUC. The top, second, third, and bottom rows for each type/grade indicate the performance on the internal validation cohort, internal testing cohort, external testing cohort 1 and 2, respectively. Five models were compared, including the proposed clustering-based model, the classical MIL model, the attention-based MIL (AMIL) model, the clustering-constraint-attention MIL (CLAM) model, and the all-patch model. Task 1: classifying the six categories of A2, A3, A4, O2, O3, and GBM. Task 2: classifying the three types of A, O, and GBM.

Task	Types/Grades	proposed	MIL	AMIL	CLAM	All-patch
1	A2	0.959	0.920	0.920	0.924	0.901
		0.970	0.913	0.915	0.910	0.843
		0.934	0.903	0.906	0.919	0.877
		0.945	0.975	0.945	0.928	0.925
	A3	0.995	0.865	0.874	0.886	0.897
		0.973	0.918	0.833	0.900	0.747
		0.923	0.933	0.917	0.959	0.755
		0.944	0.856	0.856	0.863	0.868
	A4	0.953	0.895	0.933	0.935	0.863
		0.994	0.793	0.930	0.918	0.833
		0.987	0.997	0.852	0.871	0.893
		0.904	0.892	0.844	0.890	0.895
	O2	0.978	0.956	0.923	0.928	0.912
		0.932	0.916	0.926	0.938	0.856
		0.964	0.981	0.927	0.952	0.869
		0.942	0.886	0.910	0.923	0.906
	O3	0.982	0.948	0.899	0.896	0.947
		0.980	0.945	0.925	0.899	0.909
		0.978	0.950	0.886	0.927	0.886
		0.950	0.901	0.930	0.916	0.763
	GBM	0.960	0.942	0.936	0.938	0.914
		0.980	0.953	0.949	0.951	0.910
		0.984	0.963	0.955	0.962	0.922
		0.952	0.925	0.937	0.938	0.928
2	A	0.961	0.909	0.885	0.894	0.899
		0.969	0.894	0.918	0.920	0.881
		0.938	0.897	0.932	0.930	0.846
		0.941	0.920	0.913	0.905	0.899
	O	0.974	0.960	0.921	0.955	0.919
		0.974	0.914	0.930	0.930	0.873
		0.973	0.981	0.915	0.962	0.892
		0.938	0.903	0.927	0.910	0.854
	GBM	0.960	0.942	0.936	0.938	0.914
		0.980	0.953	0.949	0.951	0.910
		0.983	0.963	0.955	0.962	0.922
		0.952	0.925	0.937	0.938	0.928

Supplementary Table 3 A summary of the P values obtained from Delong tests by comparing the AUCs between our proposed clustering-based model and each of the listed models. P value less than 0.05 indicated statistical significance. The top, second, third, and bottom rows for each type/grade indicate the P value calculated on the internal validation cohort, internal testing cohort, external testing cohort 1 and cohort 2, respectively. The AUC of the proposed clustering-based model was statistically compared with that of the listed four models by using the Delong test. The four listed models include the classical MIL model, the attention-based MIL (AMIL) model, the clustering-constraint-attention MIL (CLAM) model, and the all-patch model. Task 1: classifying the six categories of A2, A3, A4, O2, O3, and GBM. Task 2: classifying the three types of A, O, and GBM.

Task	Types/Grades	MIL	AMIL	CLAM	All-patch
1	A2	0.168	0.169	0.172	0.032
		0.031	0.060	0.043	7.3e-5
		0.119	0.596	0.788	0.208
		0.128	0.997	0.567	0.451
	A3	0.127	0.166	0.201	0.154
		0.284	0.172	0.318	0.033
		0.894	0.674	0.539	0.212
		0.079	0.217	0.403	0.072
	A4	0.183	0.687	0.729	0.050
		0.016	0.059	0.107	0.080
		0.332	0.325	0.346	0.017
		0.807	0.387	0.789	0.845
	O2	0.262	0.054	0.099	0.001
		0.595	0.686	0.759	0.134
		0.194	0.329	0.240	0.017
		0.029	0.163	0.453	0.127
	O3	0.264	0.147	0.288	0.054
		0.316	0.346	0.160	0.107
		0.314	0.192	0.321	0.062
		0.170	0.045	0.061	3.3e-4
	GBM	0.204	0.084	0.143	0.006
		0.023	0.014	0.020	2.1e-5
		0.016	0.129	0.179	0.001
		0.035	0.233	0.330	0.049
2	A	0.024	0.007	0.019	0.013
		0.005	0.014	0.029	1.8e-5
		0.028	0.764	0.820	0.022
		0.252	0.212	0.113	0.038
	O	0.379	0.046	0.117	0.002
		0.019	0.036	0.018	0.001
		0.460	0.105	0.175	0.005
		0.040	0.283	0.120	9.0e-5
	GBM	0.204	0.084	0.143	0.006
		0.023	0.014	0.020	2.1e-5
		0.016	0.129	0.178	5.6e-4
		0.035	0.232	0.330	0.0498

For classifying the six types of A2, A3, A4, O2, O3, and GBM (task 1), most P values for the three MIL models were more than 0.05, indicating that the difference in AUCs between the clustering-based model and the MIL models was not significant on most datasets. However, in most tests the AUCs of our proposed model were numerically higher than that of the MIL models. Meanwhile, we found many P values less than 0.05 for the all-patch model, indicating that the AUCs between the proposed model and the all-patch model were significantly different in many tests. For classifying the three types of A, O, and GBM (task 2), P values in almost half of tests were less than 0.05, where P values were less than 0.05 for the all-patch model in all tests.
