# Supplementary material for: Neuropathologist-level integrated classification of adult-type diffuse gliomas using deep learning from whole-slide pathological images
Source: Nat Commun. 2023 Oct 11;14:6359. doi: 10.1038/s41467-023-41195-9 (PMC10567721; doi:10.1038/s41467-023-41195-9)
Supplement: Supplementary file 3 — Description of Additional Supplementary Files [file 41467_2023_41195_MOESM3_ESM.pdf]

Description of additional Supplementary File

Supplementary Data 1: The classification performance of the MIL diagnostic model. The top, second, third and bottom rows for each type/grade indicate the performance on the internal validation cohort, internal testing cohort, and external testing cohort 1 and 2, respectively. Task 1: classifying the six categories. Task 2: classifying the three types. Task 3: classifying IDH-wildtype diffuse astrocytic tumors and IDH-mutant astrocytoma grade 2 and 3. Task 4: classifying IDH-mutant GBM in 2016 WHO classification (classified as IDH-mutant A4 in 2021 rule) and IDH-wildtype GBM. Task 5-6: classifying grades within types. Task 7: classifying IDH-mutant A and IDH-mutant 1p/19q-codeleted O.

Supplementary Data 2: The classification performance of the all-patch diagnostic model. The top, second, third and bottom rows for each type/grade indicate the performance on the internal validation cohort, internal testing cohort, and external testing cohort 1 and 2, respectively. Task 1: classifying the six categories. Task 2: classifying the three types. Task 3: classifying IDH-wildtype diffuse astrocytic tumors and IDH-mutant astrocytoma grade 2 and 3. Task 4: classifying IDH-mutant GBM in 2016 WHO classification (classified as IDH-mutant A4 in 2021 rule) and IDH-wildtype GBM. Task 5-6: classifying grades within types. Task 7: classifying IDH-mutant A and IDH-mutant 1p/19q-codeleted O.
